# Cholecystokinin and Panic Disorder: Reflections on the History and Some Unsolved Questions

**DOI:** 10.3390/molecules26185657

**Published:** 2021-09-17

**Authors:** Jens F. Rehfeld

**Affiliations:** Department of Clinical Biochemistry, Rigshospitalet, University of Copenhagen, DK-2100 Copenhagen, Denmark; jens.f.rehfeld@regionh.dk; Tel.: +45-3545-3018

**Keywords:** anxiety, cholecystokinin (CCK), neuropeptides, panic disorder, panicogenicity, peptidergic neurotransmission

## Abstract

The classic gut hormone cholecystokinin (CCK) and its CCK_2_-receptor are expressed in almost all regions of the brain. This widespread expression makes CCK by far the most abundant peptidergic transmitter system in the brain. This CNS-ubiquity has, however, complicated the delineation of the roles of CCK peptides in normal brain functions and neuropsychiatric diseases. Nevertheless, the common panic disorder disease is apparently associated with CCK in the brain. Thus, the C-terminal tetrapeptide fragment of CCK (CCK-4) induces, by intravenous administration in a dose-related manner, panic attacks that are similar to the endogenous attacks in panic disorder patients. This review describes the history behind the discovery of the panicogenic effect of CCK-4. Subsequently, the review discusses three unsettled questions about the involvement of cerebral CCK in the pathogenesis of anxiety and panic disorder, including therapeutic attempts with CCK_2_-receptor antagonists.

## 1. Introduction

Cholecystokinin (CCK) is an established gut hormone that stimulates gallbladder contraction and the emptying of bile into the small intestine [1], hence its name. CCK also regulates pancreatic enzyme secretion and growth, and it influences gastric emptying, intestinal motility, and satiety (for reviews, see [2,3,4]). On top of the essential gastrointestinal and pancreatic functions, the late 1970s showed—at that time surprisingly—that CCK is also a major neuropeptide in the central and peripheral nervous system [5,6,7,8,9,10].

The cerebral expression is unique among neuropeptides, in the sense that CCK in adult mammalian brains is abundantly present in all regions, except the cerebellum [9,10]. Moreover, the tissue concentrations, not least in the neocortical regions, are significantly higher than those measured for other neuropeptides [9,11]. In addition, the total amounts of CCK synthesized in the human brain are beyond those of the gut. CCK peptides have also turned out to be potent neurotransmitters [12,13]. As in the intestinal endocrine I-cells, CCK peptides in cerebral neurons mature from proCCK processing to different bioactive forms. CCK neurons have the short O-sulfated CCK-8 as the predominant form [6,9,14] and the shorter, nonsulfated CCK-5 as the second most abundant form [15,16]. In contrast, intestinal I-cells mainly release longer molecular forms (CCK-58, -33, and -22 in sulfated, as well as nonsulfated, variants [17,18,19]). The molecular size differences between the CCK peptides in endocrine gut cells and cerebral neurons are governed by the expression of prohormone convertases (PCs) along the secretory pathway within the cells. The endocrine I-cells mainly utilize PC 1/3, whereas CCK-neurons primarily use PC 2 in the cellular processing [20].

When it comes to the association of CCK with panic disorder, with a lifetime prevalence of 3.5% (5% in women and 2% in men) [21], a couple of observations are worth remembering. First, among brain regions in mammals, the amygdala plays a decisive role in fear and anxiety [22,23,24]. Accordingly, the centromedial amygdaloid nuclei are also particularly rich in networks of CCK neurons [10,25,26] and express high densities of CCK_2_-receptors [27,28,29]. Second, although CCK-8 in its sulfated form is the predominant cerebral form of CCK, and may by exogenous administration (of CCK-8S itself or its analogue, caerulein) cause anxiety and panic attacks [24,30,31], the short C-terminal tetrapeptide fragment, CCK-4, appears on a molar basis to be a more potent panicogenic peptide [24,30]. These observations and other unsettled questions will be discussed in the following.

## 2. The Bioactivity of CCK-4

**Figure 1:**
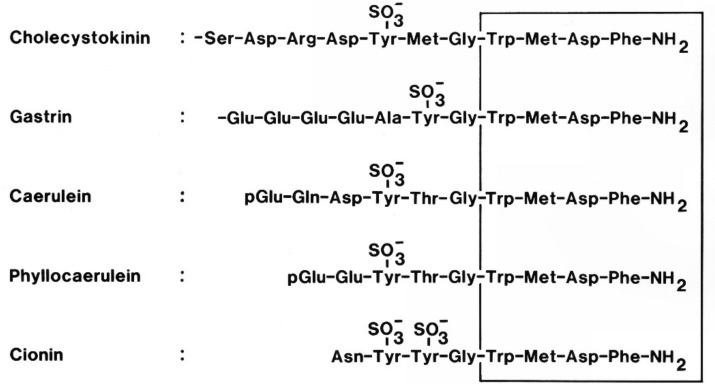
The C-terminal amino acid sequences of members of the cholecystokinin family.

The interest in the biological activity of the carboxyamidated tetrapeptide of CCK (Trp-Met-Asp-Phe·CONH_2_) dates back to the structural identification of another gastrointestinal hormone, the gastric acid regulatory hormone, gastrin [32,33]. Detailed structure–function studies of the identified gastrin-17 peptide revealed that the “active site” or receptor epitope of gastrin was precisely confined to the C-terminal tetrapeptide amide [34]. Thus, substitution of any of the four amino acid residues in the tetrapeptide (Trp, Met, Asp, Phe) reduced the acid-stimulatory activity markedly. A few years later, identification of the structure of intestinal CCK-33 showed a remarkable homology between gastrin-17 and CCK-33 [35,36]. Thus, CCK has exactly the same C-terminal bioactive tetrapeptide sequence as gastrin (Figure 1). Moreover, this common carboxyamidated sequence has been exceedingly well preserved during 600 million years of evolution [37,38,39,40], a fact that emphasizes the biological significance of the common tetrapeptide.

The discovery that the activity of gastrin could be mimicked by the smaller C-terminal tetrapeptide fragment in the 1960s [34] had two immediate consequences: First, the tetrapeptide was a considerably cheaper secretagogue to synthetize in comparison with the full-length gastrin-17 peptide, and gastrin was needed for clinical and basic studies of gastric acid secretion. The context in those years was that gastrin and its effect on acid secretion was essential for understanding the pathophysiology of the widespread duodenal ulcer disease. Along that line, the so-called “Pentagastrin^®^” was also synthetized. “Pentagastrin” is, however, a misnomer for a synthetic analogue with a BOC-Ala group coupled to the N-terminus of the authentic tetragastrin sequence (Figure 1). Second, some pharmaceutical companies synthesized tetragastrin analogues as possible drug targets for development of a gastrin receptor antagonist, to be used in the treatment of the gastric acid hypersecretion in duodenal ulcer patients that otherwise required major gastric surgery. However, a useful gastrin-4 based drug never materialized, and some pharmaceutical companies therefore had stocks of unused synthetic tetragastrin (alias CCK-4). One of these companies was the Danish LEO Pharmaceutical Products, who had prepared thousands of ampoules, each containing 70 µg of the tetrapeptide, to be injected as a bolus in dyspeptic ulcer patients for measurement of the gastric acid output.

In 1968, I wanted to study the effect of gastrin on insulin secretion after the rediscovery of incretin (incretin is the gut hormonal stimulation of insulin secretion [41,42] (for a recent review, see [43])). With limited funding, a generous gift of gastrin-4 ampoules from LEO Pharma was an appreciated help. Indeed, a bolus injection of gastrin-4 in healthy young men released considerably more insulin than a gastrin-17 preparation, in spite of similar effects on gastric acid secretion [44,45]. The potent insulinogenic effect of the tetrapeptide was later confirmed in more comprehensive studies using an isolated, perfused porcine pancreas [46].

Since gastrin-4 (identical with CCK-4) is a potent hormone releaser, since we had plenty of the tetrapeptide from LEO Pharma, and since we had observed CCK-neurons in the hypothalamus innervating the pituitary [10], we also wished to examine the effect of the tetrapeptide of growth hormone secretion. In a pilot experiment, I consequently injected a bolus of gastrin-/CCK-4 intravenously into myself and a colleague (Dr. Thue W. Schwartz). The effect on growth hormone secretion was small, but the “side” effect was dramatic.

## 3. The Panicogenic Activity of CCK-4

Half a minute after the injection of CCK-4, we experienced the beginning of what was to be a full-blown panic attack. The symptoms were intense anxiety, with a fear of dying, and a strange sense of the world sliding away, accompanied by palpitations, sweating, and faintness. The attack peaked after 5–8 min, and then gradually disappeared during the following 15–20 min [47]. None of us had experienced such an attack before. Of course, we wanted to follow it up. However, shortly after, my colleague moved to Chicago, and I got a busy chair in Copenhagen. In July 1984, however, Vanderhaeghen and Crawley organized an international conference on “Neuronal Cholecystokinin” in Brussels [48]. The last session of the conference was entitled “Clinical significance of neuronal cholecystokinin”. The chairman asked in the general discussion whether anyone in the audience beyond the session-speakers had observations of clinical interest. I therefore described the experienced panic attacks provoked by CCK-4. Present in the audience were also Jacques Bradwejn and Claude de Montigny from Montreal in Canada. In microiontophoretic studies in rat hippocampus they had recently observed that benzodiazepine antagonized CCK-8 (s)-induced activation of hippocampus neurons [49,50]. After the session, they contacted me about the CCK-4/panic story. I gave them information about further details of dose, injection, and symptoms. Working in psychiatry, they have since followed this up; first with systematic CCK-4 studies in healthy volunteers and patients with panic disorders [51,52,53]. Moreover, Bradwejn et al. subsequently performed dose ranging studies [54], showing that panic disorder patients have an increased sensitivity to CCK-4, and examined the therapeutic effect of CCK_2_-receptor antagonists (also including L-365, 260) [55]. Since then, the volume of clinical and experimental publications about CCK-4 and panic disorder has grown overwhelmingly, as also reflected in later reviews [56,57,58,59]. There is no doubt that Jacques Bradwejn played a leading role in this development.

Today, nobody questions CCK-4 as a robust panicogenic peptide, that is and has been a reliable tool in the study of panic disorder in man, and anxiety in most mammals. It is also well-established that CCK-4, of course, targets the cerebral CCK_2_-receptor and interacts in the provocation of anxiety with other neurotransmitter systems, such as the benzodiazepine/GABA complex, corticotropin-releasing factor, dopamine, noradrenaline, opioids, and serotonin [60,61,62,63,64,65,66,67]. Moreover, exogenous CCK-4 stimulates blood-flow in the anterior cingulate gyrus, the claustrum-insular-amygdala region, and the cerebellar vermis [68,69], and may act also at locus coeruleus and brainstem nuclei nucleus tractus solitarius, medulla, and parabranchial nuclei [70].

Regarding the CCK_2_-receptor binding, there is evidence that the receptor in patients with panic disorder may be more sensitive to CCK-4 and its analogue, pentagastrin [71,72,73]. Some genetic studies have also found variations in the genes encoding CCK and the CCK_2_-receptor [74,75,76,77,78,79].

So far, the use of synthetic CCK-4 in a high dose administered as an intravenous bolus (as described above) has been decisive for initiating the broad spectrum of investigations of the association between cerebral CCK and the widespread panic disorder in man. Moreover, a multitude of experimental anxiety-studies, not least in rodents, have used the CCK-4 approach. You might even ask whether significant knowledge about the CCK-panic disorder link would exist today without the serendipitously discovered effect of CCK-4.

## 4. Does the Brain Synthesize CCK-4 as a Separate Neuropeptide?

As mentioned above, the entirely dominant CCK-neurotransmitter is sulfated CCK-8 [9,14,16]. Beyond CCK-8 and small amounts of the longer forms (sulfated and nonsulfated CCK-58, -33, and -22 [15,80,81]), cerebral neurons, however, also synthesize the short CCK-5 (Gly-Trp-Met-Asp-Phe·NH_2_) in significant amounts [15,82]. However, an additional genuine neuronal synthesis of CCK-4 as such is questionable for different reasons: First, although the CCK-4 peptide has indeed been chemically identified in extracts of the porcine brain [15], the tissue concentrations were very low. Hence, the observed trace amounts of CCK-4 might well be a degradation product of CCK-5 occurring during extraction. CCK-4 differs from CCK-5 only by a single N-terminal glycyl residue (Figure 1). Second, so far no laboratory has managed to raise antibodies specific for the N-terminus of CCK-4. Such antibodies might by immunocytochemistry be decisive for the discussion of whether biosynthesis of CCK-4, as such, occurs in neurons. A third consideration concerns the N-terminal Trp-residue in CCK-4. The identified G-protein-coupled receptor, GPR142 [83], binds free tryptophan and to some extent also short peptides with an N-terminal Trp-residue. GPR142 is expressed in large amounts on pancreatic islet cells [84], where it seems to balance the effect of some gut hormones, including CCK, on insulin secretion [85]. Since CCK-4 more potently releases insulin than CCK-5 and the longer CCK-peptides [46], a mechanism might be that high dosing of exogenous CCK-4 simultaneously activates both the CCK_2_-receptor and GPR142 in pancreatic islets [84,86]. Whether the striking panicogenic potency of exogenous CCK-4 is due to a similar mechanism remains to be shown. If so, the lack of intracellular processing of CCK-5 to CCK-4 might make sense as a way to prevent severe panic attacks and anxiety.

## 5. Is Endogenous CCK in Plasma Associated with Panic Disorder?

CCK was, as mentioned, discovered as a gallbladder emptying gut hormone nearly a century ago [1]. Today, we know that the entire small intestine produces CCK in response to meals. We also know that essentially all the hormonally active CCK in plasma is derived from the CCK-producing endocrine I-cells in the gut [18]. Thus, even though endocrine cells in the pituitary, the thyroid C-cells, the adrenal medulla, and the testes express modest amounts of CCK [87,88,89,90,91], the extraintestinal endocrine cells contribute only marginally to the CCK in plasma.

The concentrations of bioactive CCK in mammalian plasma are low. In the fasting state, the concentrations vary from 0.1 to 2.0 pmol/L, and after a normal mixed meal, the concentrations increase to 5–7 pmol/L [92,93]. The molecular forms of CCK in human plasma are sulfated CCK-58, -33, -22, and -8, of which CCK-33 is the predominant form [18]. It is unlikely, but remains to be examined, whether any of these forms in their low picomolar plasma concentrations are able to penetrate the blood–brain barrier to any significant extent. Hence, the discrepancy with the peak in the high nanomolar range of CCK-4 concentrations in plasma after an intravenous bolus injection of 70 µg CCK-4 is enormous. It is, however, well known that sulfated CCK peptides in plasma, during and after a meal, signal satiety to the hypothalamus via CCK_1_-receptors on afferent vagal neurons [94,95]. Whether a similar neuronal mechanism, from peripheral plasma CCK to anxiety centers in the brain (amygdala), exists also remains to be studied.

## 6. CCK_2_-Receptor Antagonists in Panic Disorder Therapy

The realization that cerebral CCK_2_-receptors are the targets of CCK-4 in its induction of panic attacks clearly suggested examining receptor antagonists as therapeutic possibilities. So far, only a few antagonists have been studied. Interestingly, the well-established CCK_2_-receptor antagonist, L-365,260, reduced the panic response to the bolus of CCK-4 in patients suffering from panic disorders [55]. However, the reduction was observed only when L-365,260 was administered acutely, just before the injection of CCK-4. Placebo-controlled clinical trials showed no effect of L-365,260; presumably due to the limited bioavailability of the antagonist [96]. Along the same line, the CI-988 antagonist had only a weak effect on CCK-4-induced panic in healthy volunteers [97], but failed to affect patients with panic disorder [98]. In a recent study of anxiety in rats [99], the CCK_2_-receptor antagonist, LY-225,910, prevented the normalization of anxiety levels in rats. However, whether this antagonist also prevents attacks in panic disorder patients remains to be shown.

Generally, there is a problem in using systemic administration of CCK_2_-receptor antagonist in the therapy of panic disorder and other neuropsychiatric disorders, because CCK peptides and CCK_2_-receptors are so abundantly expressed in almost all regions of the brain [27,28,29,100] and not only in limbic structures, such as the amygdala. The blocking of the CCK-system (agonists and receptors) in the brain may have several serious effects, as shown in CCK knock-out mice, who apparently lose their memory (Jufang He, personal communication); and what is life without memory? At present, it seems difficult to overcome that kind of problem. Perhaps local administration of CCK_2_-receptor antagonists exclusively to the amygdala might help?

## 7. Conclusions

CCK peptides and the CCK_2_-receptor are, as mentioned, expressed at high levels in most brain regions, with marked densities in the cerebral cortex and limbic structures [9,10,27,28,29,100,101]. Since panic disorder is a rather common psychiatric condition, with a prevalence among women of 5% and 2% in men [21], you could argue that some involvement of the cerebral CCK-system in the pathogenesis of panic attacks should not come as a surprise. Nevertheless, it was unexpected that a simple intravenous bolus injection of the C-terminal tetrapeptide fragment of CCK should open the gates for decades of comprehensive studies of the role of CCK in panic disorder.

CCK-4 studies have unequivocally demonstrated that cerebral CCK-peptides and CCK_2_-receptors play a significant role in panic attacks. The studies have also shown that CCK-4 in high doses is a highly useful and reliable agent for provocation of panic attacks, both in healthy volunteers and panic disorder patients. In addition to CCK, however, it is now established that several other transmitter systems also are involved in the pathophysiology of panic attacks.

The story of CCK and panic disorders, however, has left several open questions. Among these, the present review has focused on three: Is CCK-4 a naturally occurring endogenous agonist for the cerebral CCK_2_-receptor? Second, is peripheral intestinal CCK in plasma associated with panic disorder? Finally, are CCK_2_-receptor antagonists useful drugs for panic disorder patients? This review suggests that CCK-4 as such, in contrast to CCK-5, can hardly be considered a natural product of biosynthesis in cerebral CCK neurons. Moreover, there is probably no association between the low CCK concentrations in plasma and panic disorder. Finally, no CCK-receptor antagonist has proved useful for therapy of panic disorder. Consequently, it is to be concluded that further basic and clinical research is necessary to answer these still unsettled questions.

## Data Availability

Not Applicable.

## References

[1] Ivy A.C., Oldberg E. (1928). Hormone mechanism for gallbladder contraction and evacuation. Am. J. Physiol..

[2] Rehfeld J.F., Schultz S.G., Makhlouf G.M., Rauner B.B. (1989). Cholecystokinin. Handbook of Physiology, the Gastrointestinal System: Neural and Endocrine Biology.

[3] Rehfeld J.F. (2017). Cholecystokinin—From local gut hormone to ubiquitous messenger. Front. Endocrinol..

[4] Rehfeld J.F. (2021). Cholecystokinin and the hormone concept. Endocr. Connect..

[5] Vanderhaeghen J.J., Signeau J.C., Gepts W. (1975). New peptide in the vertebrate CNS reacting with antigastrin antibodies. Nature.

[6] Dockray G.J. (1976). Immunochemical evidence of cholecystokinin-like peptides in brain. Nature.

[7] Müller J.E., Strauss E., Yalow R.S. (1977). Cholecystokinin and its COOH-terminal octapeptide in the pig brain. Proc. Natl. Acad. Sci. USA.

[8] Rehfeld J.F. (1977). Gastrins and cholecystokinins in brain and gut. Acta Pharmacol. Toxicol..

[9] Rehfeld J.F. (1978). Immunochemical studies on cholecystokinin. II. Distribution and molecular heterogeneity in the central nervous system and the small intestine of man and hog. J. Biol. Chem..

[10] Larsson L.-I., Rehfeld J.F. (1979). Localization and molecular heterogeneity of cholecystokinin in the central and peripheral nervous system. Brain Res..

[11] Crawley J.N. (1985). Comparative distribution of cholecystokinin and other neuropeptides: Why is this peptide different from all other peptides?. Ann. N. Y. Acad. Sci..

[12] Dodd J., Kelly J.S. (1979). Cholecystokinin peptides: Excitatory effect on hippocampal neurons. J. Physiol..

[13] Emson P.C., Lee C.M., Rehfeld J.F. (1980). Cholecystokinin octapeptide: Vesicular localization and calcium dependent release from rat brain in vitro. Life Sci..

[14] Dockray G.J., Gregory R.A., Hutchison J.B., Harris J.I., Runswick M.J. (1978). Isolation, structure and biological activity of two cholecystokinin octapeptides from sheep brain. Nature.

[15] Rehfeld J.F., Hansen H.F. (1986). Characterization of preprocholecystokinin products in porcine cerebral cortex: Evidence of different processing pathways. J. Biol. Chem..

[16] Agersnap M., Zhang M.-D., Harkany T., Hökfelt T., Rehfeld J.F. (2016). Nonsulfated cholecystokinins in cerebral neurons. Neuropeptides.

[17] Eberlein G.A., Eysselein V.E., Davis M.T., Lee T.D., Shively J.E., Grandt D., Niebel W., Williams R., Moessner J., Zeeh J. (1992). Patterns of prohormone processing: Order revealed by a new procholecystokinin-derived peptide. J. Biol. Chem..

[18] Rehfeld J.F., Sun G., Christensen T., Hillingsø J.G. (2001). The predominant cholocystokinin in human plasm and intestine is cholecystokinin-33. J. Clin. Endocrinol. Metab..

[19] Agersnap M., Rehfeld J.F. (2015). Nonsulfated cholecystokinins in the small intestine of pigs and rats. Peptides.

[20] Rehfeld J.F., Bundgaard J.R., Hannibal J., Zhu X., Norrbom C., Steiner D.F., Friis-Hansen L. (2008). The cell-specific pattern of cholecystokinin peptides in endocrine cells versus neurons is governed by the expression of prohormone convertases 1/3, 2, and 5/6. Endocrinology.

[21] Kessler R.C., McGonagle K.A., Zhao S., Nelson C.B., Hughes M., Eshleman S., Wittchen H.U., Kendler K.S. (1994). Lifetime and 12-month prevalence of DSM-III-R psychiatric disorders in the United States. Results from the national Comorbidity Survey. Arch. Gen. Psychiatry.

[22] LeDoux J.E. (2000). Emotion circuits in the brain. Annu. Rev. Neurosci..

[23] Davis M., Whalen P.J. (2001). The amygdala: Vigilance and emotion. Mol. Psychiatry.

[24] Pérez de la Mora M., Hernández-Gómez A.M., Arizmendi-Garcia Y., Jacobsen K.X., Lara-Garcia D., Flores-Gracia C., Crespo-Ramirez M., Gallegos-Cari A., Nuche-Bricaire A., Fuxe K. (2007). Role of the amygdoloid cholecystokinin (CCK)/gastrin-2 receptors and terminal networks in the modulation of anxiety in the rat. Effects of CCK-4 and CCK-8S on anxiety-like behaviour and [^3^H] GABA release. Eur. J. Neurosci..

[25] Vanderhaeghen J.J., Lotstra F., De Mey J., Gilles C. (1980). Immunohistochemical localization of cholecystokinin- and gastrin-like peptides in the brain and hypophysis of the rat. Proc. Natl. Acad. Sci. USA.

[26] Mascagni F., McDonald A.J. (2003). Immunohistochemical characterization of cholecystokinin containing neurons in the rat basolateral amygdala. Brain Res..

[27] Zarbin M.A., Innis R.B., Wamsley J.K., Snyder S.H., Kuhar M.J. (1983). Autoradiographic localization of cholecystokinin receptors in rodent brain. J. Neurosci..

[28] Honda T., Wada E., Battey J.F., Wank S.A. (1993). Differential expression of CCK(A) and CCK(B) receptors in the rat brain. Mol. Cell. Neurosci..

[29] Mercer L.D., Le V.Q., Nunan J., Jones N.M., Beart P.M. (2000). Direct visualization of cholecystokinin subtype2 receptors in rat central nervous system using antipeptide antibodies. Neurosci. Lett..

[30] Rex A., Barth T., Voigt J.P., Domeney A.M., Fink H. (1994). Effects of cholecystokinin tetrapeptide and sulfated cholecystokinin octapeptide in rat models of anxiety. Neurosci. Lett..

[31] Köks S., Männistö P.T., Bourin M., Shlik J., Vasar V., Vasar E. (2000). Cholecystokinin-induced anxiety in rats: Relevance of pre-experimental stress and seasonal variations. J. Psychiatry Neurosci..

[32] Gregory R.A., Tracy H.J. (1964). The constitution and properties of two gastrins extracted from hog antral mucosa. Gut.

[33] Gregory H., Hardy P.M., Jones D.S., Kenner G.W., Sheppard R.C. (1964). The antral hormone gastrin. I. Structure of gastrin. Nature.

[34] Morley J.S., Tracy H.J., Gregory R.A. (1965). Structure-function relationships in the active C-terminal tetrapeptide sequence of gastrin. Nature.

[35] Mutt V., Jorpes J.E. (1968). Structure of porcine cholecystokinin—Pancreozymin. I. Cleavage with thrombin and with trypsin. Eur. J. Biochem..

[36] Mutt V., Jorpes J.E. (1971). Hormonal polypeptides of the upper intestine. Biochem. J..

[37] Larsson L.-I., Rehfeld J.F. (1977). Evidence for a common evolutionary origin of gastrin and cholecystokinin. Nature.

[38] Johnsen A.H. (1998). Phylogeny of the cholecystokinin/gastrin family. Front. Neuroendocrinol..

[39] Baldwin B.S., Patel O., Shulkes A. (2010). Evolution of gastrointestinal hormones: The cholecystokinin/gastrin family. Curr. Opin. Endocrinol. Diabetes Obes..

[40] Dupré D., Tostivint H. (2014). Evolution of the gastrin-cholecystokinin gene family by synteny analysis. Gen. Comp. Endocrinol..

[41] Elrick H., Stimmler L., Hlad C.J., Arai Y. (1964). Plasma insulin responses to oral and intravenous glucose administration. J. Clin. Endocrinol. Metab..

[42] McIntyre N., Holdsworth C.D., Turner D.A. (1964). New interpretation of oral glucose tolerance. Lancet.

[43] Rehfeld J.F. (2018). The origin and understanding of the incretin concept. Front. Endocrinol..

[44] Rehfeld J.F. (1971). Effect of gastrin and its C-terminal tetrapeptide on insulin secretion in man. Acta Endocrinol..

[45] Rehfeld J.F., Stadil F. (1973). The effect of gastrin on basal- and glucose-stimulated insulin secretion in man. J. Clin. Investig..

[46] Rehfeld J.F., Larsson L.-I., Goltermann N.R., Schwartz T.W., Holst J.J., Jensen S.L., Morley J.S. (1980). Neural regulation of pancreatic hormone secretion by the C-terminal tetrapeptide of CCK. Nature.

[47] Rehfeld J.F., Dourish C.T., Cooper S.J., Iversen S.D., Iversen L.L. (1992). CCK and anxiety: An introduction. Multiple Cholecystokinin Receptors in CNS.

[48] Vanderhaeghen J.J., Crawley J.N. (1985). Neuronal cholecystokinin. International Conference on Neuronal Cholecystokinin 1984: Brussels, Belgium.

[49] Bradwejn J., de Montigny C. (1984). Benzodiazepine antagonizes cholecystokinin-induced activation of rat hippocampal neurons. Nature.

[50] Bradwejn J., de Montigny C. (1985). Antagonism of cholecystokinin-induced activation by benzodiazepine receptor antagonists. Ann. N. Y. Acad. Sci..

[51] de Montigny C. (1989). Cholecystokinin tetrapeptide induces panic-like attacks in healthy volunteers: Preliminary findings. Arch. Gen. Psychiatry.

[52] Bradwejn J., Koszycki D., Meterissian G. (1990). Cholecystokinin tetrapeptide induces panic attacks in patients with panic disorders. Can. J. Psychiatry.

[53] Bradwejn J., Koszycki D., Shriqui C. (1991). Enhanced sensitivity to cholecystokinin tetrapeptide in panic disorder. Arch. Gen. Psychiatry.

[54] Bradwejn J., Koszycki D., Bourin M. (1991). Dose-ranging study of the effect of CCK-4 in healthy volunteers. J. Psychiatr. Neurosci..

[55] Bradwejn J., Koszycki D., Couëtoux du Tertre A., van Megen H., den Boer J., Westenberg H. (1994). The panicogenic effects of cholecystokinin tetrapeptide are antagonized by L-365,260, a central cholecystokinin receptor antagonist, in patients with panic disorder. Arch. Gen. Psychiatry.

[56] Bradwejn J., Vasar E. (1995). Cholecystokinin and Anxiety: From Neuron to Behavior.

[57] Bradwejn J., Koszycki D. (2001). Cholecystokinin and panic disorder: Past and future clinical research strategies. Scand. J. Clin. Lab. Investig..

[58] Wang H., Wong P.T.H., Spiess J., Zhu Y.Z. (2005). Cholecystokinin-2 (CCK_2_) receptor mediated anxiety-like behaviors in rats. Neurosci. Biobehav. Rev..

[59] Zwanzger P., Domschke K., Bradwejn J. (2012). Neuronal network of panic disorder: The role of the neuropeptide cholecystokinin. Depress. Anxiety.

[60] McCann U.D., Slate S.O., Geraci M., Roscow-Terrill D., Uhde T.W. (1997). A comparison of the effects of intravenous pentagastrin on patients with social phobia, panic disorder and healthy controls. Neuropsychopharmacology.

[61] Koszycki D., Zacharko R., Le Mellédo J.M., Bradwejn J. (1998). Behavioural, cardiovascular and neuroendocrine profiles following CCK-4 challenge in healthy volunteers: A comparison of panickers and non-panickers. Depress. Anxiety.

[62] Shlik J., Aluoja A., Vasar V., Vasar E., Podar T., Bradwejn J. (1997). Effects of citapram treatment on behavioural, cardiovascular, and neuroendocrine response to cholecystokinin tetrapeptide challenge in panic disorder patients. J. Psychiatry Neurosci..

[63] van Megen H.J., Westenberg G.M., den Boer J.A., Slaap B., Scheepmakers A. (1997). Effect of the selective serotonin reuptake inhibitor fluvoxamine on CCK-4 induced panic attacks. Psychoparmacology.

[64] Zacharko R.M., Koszycki D., Mendella P.D., Bradwejn J. (1995). Behavioral, neurochemical, anatomical and electrophysiological correlates of panic disorder: Multiple transmitter interaction and neuropeptide colocalizations. Prog. Neurobiol..

[65] Crawley J.N., Bradwejn J., Vasar E. (1995). Interactions between cholecystokinin and other neurotransmitter systems. Cholecystokinin and Anxiety: From Neuron to Behavior.

[66] Koszycki D., Zacharko R., Le Melledo J.M., Young S.N., Bradwejn J. (1996). Effect of acute tryptophan depletion on behavioural, cardiovascular and hormonal sensitivity to cholecystokinin-tetrepeptide challenge in healthy volunteers. Biol. Psychiatry.

[67] Le Mellédo J.M., Bradwejn J., Koszycki D., Bichet D.G., Ballavance F. (1998). The role of the beta-noradrenergic system in cholecystokinin-tetrapeptide-induced panic symptoms. Biol. Psychiatry.

[68] Benkelfat C., Bradwejn J., Meyer E., Ellenbogen M., Milot S., Gjedde A., Evans A. (1995). Functional neuroanatomy of CCK4-induced anxiety in normal healthy volunteers. Am. J. Psychiatry.

[69] Javanmard M., Shilk J., Kennedy S.H., Vaccarino F.J., Houle S., Bradwejn J. (1999). Neuroanatomic correlates of CCK-4-induced panic attacks in healthy humans: A comparison of two time points. Biol. Psychiatry.

[70] Denavit-Saubié M., Hurlé M.A., Morin-Surun M.P., Foutz A.S., Champagnat J. (1985). The effects of cholecystokinin-8 in the nucleus tractus solitarius. Ann. N. Y. Acad. Sci..

[71] de Leeuw A.S., den Boer J.A., Slaap B.R., Westenberg H.G. (1996). Pentagastrin has panic-inducing properties in obsessive compulsive disorder. Psychopharmacology.

[72] van Vliet I.M., Westenberg H.G., Slaap B.R., den Boer J.A., Ho Pial K.L. (1997). Anxiogenic effects of pentagastrin in patients with social phobia and healthy controls. Biol. Psychiatry.

[73] Brawman-Minzter O., Lydiard R.B., Bradwejn J., Villarreal G., Knapp R., Emmanuel N., Ware M.R., He Q., Ballenger J.C. (1997). Effects of the cholecystokinin agonist pentagastrin in patients with generalized anxiety disorder. Am. J. Psychiatry.

[74] Hansen T.V.O., Rehfeld J.F., Nielsen F.C. (2000). Function of the C-36 to T polymorphism in the human cholecystokinin gene promoter. Mol. Psychiatry.

[75] Wang Z., Valdes J., Noyes R., Zoega T., Crowe R.R. (1998). Possible association of a cholecystokinin promotor polymorphism (CCK-36CT) with panic disorder. Am. J. Med. Genet..

[76] Garvey M.J., Crowe R.R., Wang Z. (1998). An association of NAG levels and a mutation of the CCK gene in panic disorder patients. Psychiatry Res..

[77] Koefoed P., Woldbye D.P., Hansen T.V.O., Hansen E.S., Knudsen G.M., Bolwig T.G., Rehfeld J.F. (2010). Gene variations in the cholecystokinin system in patients with panic disorder. Psychiatr. Genet..

[78] Hösing V.G., Schirmacher A., Kuhlenbäumer G., Freitag C., Sand P., Schlesiger C., Jacob C., Fritze J., Franke P., Rietschel M. (2004). Cholecystokinin- and cholecystokinin-B-receptor gene polymorphism in panic disorder. J. Neural Transmission. Suppl..

[79] Hattori E., Yamada K., Toyota T., Yoshitsugu K., Toru M., Shibuya H., Yoshikawa T. (2001). Association studies of the CT repeat polymorphism in the 5’ upstream region of the cholecystokinin B receptor gene with panic disorder and schizophrenia in Japanese subjects. Am. J. Med. Genet..

[80] Eysselein V.E., Reeve J.R., Shively J.E., Miller C., Walsh J.H. (1984). Isolation of a large cholecystokinin precursor from canine brain. Proc. Natl. Acad. Sci. USA.

[81] Tatemoto K., Jörnvall H., Siimesmaa S., Halldén G., Mutt V. (1984). Isolation and characterization of cholecystokinin-58 (CCK-58) from porcine brain. FEBS Lett..

[82] Shively J., Reeve J.R., Eysselein V.E., Ben-Avram C., Vigna S.R., Walsh J.H. (1987). CCK-5: Sequence analysis of a small cholecystokinin from canine brain and intestine. Am. J. Physiol..

[83] Süsens U., Hermans-Borgmeyer I., Urny J., Schaller H.C. (2006). Characterisation and differential expression of two very closely related G-protein-coupled receptors, GPR139 and GPR142, in mouse tissue and during mouse development. Neuropharmacology.

[84] Regard J.B., Sato I.T., Coughlin S.R. (2008). Anatomical profiling of G protein-coupled receptor expression. Cell.

[85] Rudenko O., Shang J., Munk A., Ekberg J.P., Petersen N., Engelstoft M.S., Egerod K.L., Hjorth S.A., Wu M., Feng Y. (2019). The aromatic amino acid sensor GPR142 controls metabolism through balanced regulation of pancreatic and gut hormones. Mol. Metab..

[86] Reubi J.C., Waser B., Gugger M., Friess H., Kleeff J., Kayed H., Büchler M.W., Laissue J.A. (2003). Distribution of CCK1 and CCK2 receptors in normal and diseased human pancreatic tissue. Gastroenterology.

[87] Rehfeld J.F. (1986). Accumulation of nonamidated preprogastrin and preprocholecystokinin products in porcine pituitary corticotrophs: Evidence of post-translational control of cell differentiation. J. Biol. Chem..

[88] Rehfeld J.F. (1987). Preprocholecystokinin processing in the normal human anterior pituitary. Proc. Natl. Acad. Sci. USA.

[89] Rehfeld J.F., Johnsen A.H., Ødum L., Bardram L., Schifter S., Scopsi L. (1990). Non-sulphated cholecystokinin in human medullary thyroid carcinomas. J. Endocrinol..

[90] Bardram L., Hilsted L., Rehfeld J.F. (1989). Cholecystokinin, gastrin and their precursors in pheochromocytomas. Acta Endocrinol..

[91] Persson H., Rehfeld J.F., Ericsson A., Schalling M., Pelto-Huikko M., Hökfelt T. (1989). Transient expression of the cholecystokinin gene in male germ cells and accumulation of the peptide in the acrosomal granule: Possible role of cholecystokinin in fertilization. Proc. Natl. Acad. Sci. USA.

[92] Rehfeld J.F. (1998). Accurate measurement of cholecystokinin in plasma. Clin. Chem..

[93] Rehfeld J.F., Sennels H.P., Jørgensen H.L., Fahrenkrug J. (2020). Circadian variations in plasma concentrations of cholecystokinin and gastrin in man. Scand. J. Clin. Lab. Investig..

[94] Gibbs J., Young R.C., Smith G.P. (1973). Cholecystokinin elicits satiety in rats with open gastric fistulas. Nature.

[95] Smith G.P., Jerome C., Cushin B.J., Eterno R., Simansky K.J. (1981). Abdominal vagotomy blocks the satiety effect of cholecystokinin in the rat. Science.

[96] Kramer M.S., Cutler N.R., Ballenger J.C., Patterson W.M., Mendels J., Chenault A., Shrivastava R., Matzura-Wolfe D., Lines C., Reines S. (1995). A placebo-controlled trial of L-365,260, a CCKB antagonist, in panic disorder. Biol. Psychiatry.

[97] Bradwejn J., Koszycki D., Paradis M., Reece P., Hinton J., Sedman A. (1995). Effect of CI-988 on cholecystokinin tetrapeptide-induced panic symptoms in healthy volunteers. Biol. Psychiatry.

[98] van Megen H.J., Westenberg G.M., den Boer J.A., Slaap B., van Es-Radhakishun F., Pande A.C. (1997). The cholecystokinin-B receptor antagonist CI-988 failed to affect CCK-4 induced symptoms in panic disorder patients. Psychopharmacology.

[99] Ballaz S.J., Bourin M., Akil H., Watson S.J. (2020). Blockade of the cholecystokinin CCK-2 receptor prevents the normalization of anxiety levels in the rat. Prog. Neuropsychopharmacol. Biol. Psychiatry.

[100] Innis R.B., Snyder S.H. (1980). Distinct cholecystokinin receptors in brain and pancreas. Proc. Natl. Acad. Sci. USA.

[101] Lee Y.M., Beinborn M., McBride E.W., Lu M., Kolakowski L.F., Kopin A.S. (1993). The human brain cholecystokinin-B/gastrin receptor. Cloning and characterization. J. Biol. Chem..

